# Anti-COVID-19, Anti-Inflammatory, and Anti-Osteoarthritis Activities of Sesamin from *Sesamum indicum* L.

**DOI:** 10.3390/bioengineering10111263

**Published:** 2023-10-30

**Authors:** Shu-Ming Huang, Cheng-Yang Hsieh, Jasmine U. Ting, Kathlia A. De Castro-Cruz, Ching-Chiung Wang, Chia-Jung Lee, Po-Wei Tsai

**Affiliations:** 1 Department of Nutrition, College of Medical and Health Care, Hungkuang University, Taichung 433, Taiwan; alice926112@gmail.com; 2 Department of Nutrition, Nantou Hospital of Ministry of Health and Welfare, Nantou 540, Taiwan; 3 Ph.D. Program in Clinical Drug Development of Herbal Medicine, College of Pharmacy, Taipei Medical University, Taipei 110, Taiwan; d339108001@tmu.edu.tw (C.-Y.H.); crystal@tmu.edu.tw (C.-C.W.); 4 Laboratory of Oncology, Pharmacy Practice and Sciences, Graduate School of Pharmaceutical Sciences, Tohoku University, Sendai 980-8577, Japan; 5 Department of Chemistry, College of Science, De La Salle University, Metro Manila 1004, Philippines; jasmine.u.ting@gmail.com; 6 School of Chemical, Biological, and Materials Engineering and Sciences, Mapúa University, Metro Manila 1002, Philippines; kadecastro@mapua.edu.ph; 7 Traditional Herbal Medicine Research Center, Taipei Medical University Hospital, Taipei 110, Taiwan; 8 Graduate Institute of Pharmacognosy, College of Pharmacy, Taipei Medical University, Taipei 110, Taiwan; 9 School of Pharmacy, College of Pharmacy, Taipei Medical University, Taipei 110, Taiwan; 10 Orthopedics Research Center, Taipei Medical University Hospital, Taipei 110, Taiwan; 11 Department of Medical Science Industries, College of Health Sciences, Chang Jung Christian University, Tainan 711, Taiwan

**Keywords:** anti-COVID-19, anti-inflammatory, anti-osteoarthritis, molecular docking, SARS-CoV-2 RdRp, human ACE2

## Abstract

During the COVID-19 (coronavirus disease 2019) outbreak, many people were infected, and the symptoms may persist for several weeks or months for recovering patients. This is also known as “long COVID” and includes symptoms such as fatigue, joint pain, muscle pain, et cetera. The COVID-19 virus may trigger hyper-inflammation associated with cytokine levels in the body. COVID-19 can trigger inflammation in the joints, which can lead to osteoarthritis (OA), while long-term COVID-19 symptoms may lead to joint damage and other inflammation problems. According to several studies, sesame has potent anti-inflammatory properties due to its major constituent, sesamin. This study examined sesamin’s anti-inflammatory, anti-osteoarthritis, and anti-COVID-19 effects. Moreover, in vivo and in vitro assays were used to determine sesamin’s anti-inflammatory activity against the RAW264.7 and SW1353 cell lines. Sesamin had a dose-dependent effect (20 mg/kg) in a monoiodoacetic acid (MIA)-induced osteoarthritis rat model. Sesamin reduced paw swelling and joint discomfort. In addition, the findings indicated that sesamin suppressed the expression of iNOS (inducible nitric oxide synthase) and COX-2 (cyclooxygenase-2) in the RAW264.7 cell line within the concentration range of 6.25–50 μM. Furthermore, sesamin also had a suppressive effect on MMP (matrix metalloproteinase) expression in chondrocytes and the SW1353 cell line within the same concentration range of 6.25–50 μM. To examine the anti-viral activity, an in silico analysis was performed to evaluate sesamin’s binding affinity with SARS-CoV-2 RdRp (severe acute respiratory syndrome coronavirus 2 RNA-dependent RNA polymerase) and human ACE2 (angiotensin-converting enzyme 2). Compared to the controls, sesamin exhibited strong binding affinities towards SARS-CoV-2 RdRp and human ACE2. Furthermore, sesamin had a higher binding affinity for the ACE2 target protein. This study suggests that sesamin shows potential anti-SARS-CoV-2 activity for drug development.

## 1. Introduction

The worldwide COVID-19 outbreak led to 607.7 million confirmed cases and 6.4 million deaths (recorded by the WHO) until November 2022 [1]. The most common acute symptoms of COVID-19 patients (4–5 days after exposure) include a cough, sputum, shortness of breath, and a fever [2,3]. Patients who have recovered from COVID-19 may still experience long-term symptoms such as fatigue, breathlessness, a cough, palpitations, headaches, joint pain, and muscle pain, which can persist for weeks or months. Patients have displayed hypocalcemia and hypovitaminosis D, leading to bone demineralization while also having joint and muscle pain, the same process as early osteoarthritis due to aging [4]. Early 2020 saw a global discussion about whether NSAIDs could worsen COVID-19 symptoms. Viruses can directly attack arthritis or go through the body’s immune system, producing the host’s response to inflammation [5,6,7]. The SARS-CoV-2 infection is mediated by the binding of the viral S protein located in its receptor-binding domain (RBD) to ACE2 (angiotensin-converting enzyme 2) [8,9,10].

Studies suggest that COVID-19 patients with rheumatoid arthritis or osteoarthritis treated with NSAIDs have a lower risk of death. In a randomized study, researchers discovered that COVID-19 patients manifested hypovitaminosis D and a significantly higher risk of death [11]. For this, bone demineralization is an observed consequence due to prolonged bed rest and is associated with quarantines, vitamin D deficiency, and hypocalcemia. Vitamin D has a major role to play in regulating the immune system balance [4,12,13]. After six months, a COVID-19 infection causes considerable joint pain in the knees, shoulders, ankles, and other body parts. MRI failed to detect damage in these patients with neurogenic or low-grade synovitis. They experienced non-specific joint pain for two to four years [14]. The evidence shows that after a COVID-19 infection, myalgia is associated with the pain in the acute phase of a COVID-19 infection that does not resolve and becomes persistent musculoskeletal pain, with long periods of COVID-19 symptoms [15,16,17,18,19].

Due to age, joint overuse, and obesity, osteoarthritis (OA) is a chronic articular inflammatory disease that dilapidates the joints and tears down the cartilage matrix [20,21,22,23]. In addition, lipopolysaccharides are other factors that induce inflammation and are commonly found in the outer membranes of Gram-negative bacteria [22]. In the progression of osteoarthritis, pro-inflammatory cytokines such as TNF-α and interleukins can stimulate the production of matrix metalloproteinases (MMPs) via the c-Fos signaling pathway and other pro-inflammatory signals such as PGE2, NO, COX-2, and iNOS in chondrocytes [24,25,26]. In addition, the denaturation of type II collagen and proteoglycan caused by MMPs can weaken the cartilage matrix due to the increase in the water content [27,28]. Therefore, extracellular matrix (ECM) cartilage degradation and synthesis are imbalanced.

In Asian cuisine, sesame is one of the oldest condiments, and it was the first crop used to produce oil from its seeds [29,30,31,32]. As early as the 8th century B.C., Chinese and Indian people discovered the medicinal value of sesame seeds [32,33,34], and they believed that sesame possessed properties for the abatement of toothaches, energy recovery, rejuvenation, relaxation, and the treatment of insect bites. Due to the economic and medicinal value of the seeds, 70% of the world’s planting area produces and cultivates sesame seeds (which accounts for 60% of the global production). The sesame producers are mostly found in tropical countries in Africa and Asia (e.g., Myanmar, India, China, Sudan, and Ethiopia) [33,35,36]. Sesame seeds contain about 50% oil, 25% protein, and other components [37,38]. Among all the components, sesame lignans (especially sesamin and sesamolin) have been shown to possess various pharmacological activities [39]. In experimental animal models, sesame oil was found to have analgesic, antipyretic, and anti-inflammatory properties. In addition, sesame oil has been found to mitigate nonalcoholic fatty liver disease in patients and lower left ventricular hypertrophy in rats. Recent studies have shown that sesamin can improve the lipid metabolism of fatty livers, inhibit pro-inflammatory factors in rats with acute hepatic injury, decrease blood glucose levels and suppress microglia activation in a mouse model of diabetic retinopathy, remove free radicals, and inhibit lipid peroxidation [40,41,42].

To assess a drug’s anti-arthritic property, several models, such as the mono-iodoacetate (MIA)-induced osteoarthritis (OA) model, have been employed to replicate the pathological changes observed in OA. When administered intra-articularly, MIA induces inflammatory responses and disrupts the metabolic processes of chondrocytes. The disruption in chondrocytes has the potential to induce the deterioration of the cartilage matrix. The present study aimed to examine the possible impact of sesamin on osteoarthritis (OA) utilizing an in vivo OA model. The mechanism of COVID-19 infection involves the interaction between the receptor-binding domain (RBD) of the viral S protein and ACE2 [43]. Hence, a molecular docking approach was employed to examine the potential anti-COVID-19 properties of sesamin in relation to pharmacological compounds that target the RdRp of SARS-CoV-2, serving as anti-viral markers. This first-of-its-kind anti-viral activity screening research focused on the screening of sesamin’s pharmacological properties, including its potential as an anti-inflammatory, anti-osteoarthritis, and anti-COVID-19 agent. The primary aim of our study was to develop a drug that exhibits both anti-osteoarthritis and anti-COVID-19 properties, with the additional goal of mitigating symptoms in individuals afflicted with COVID-19.

## 2. Materials and Methods

### 2.1. Reagent

**Figure 1:**
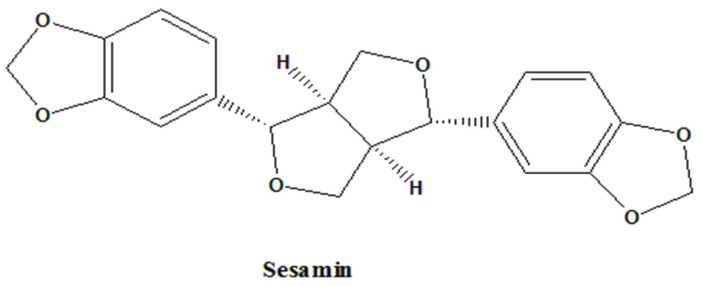
Chemical structures of sesamin.

All chemicals, including sesamin (Figure 1), were purchased from Sigma (St. Louis, MO, USA) through Uni-Onward Corp., New Taipei City, Taiwan.

### 2.2. Anti-Inflammatory Assay

#### 2.2.1. Rat Chondrocyte Primary Culture

The articular surface cartilage was extracted from both knee joints of mature male Wistar rats procured from LASCO Co., Ltd. (Taipei City, Taiwan) A Pronase solution with a concentration of 10 mg/mL and a volume of 20 mL was employed to treat cartilage that had been shaved in a dish for a duration of 30 min at a temperature of 37 °C. Subsequently, 5 mg/mL collagenase IV was introduced into the dish, and all specimens were subjected to incubation at a temperature of 37 °C for a duration of 4 h, during which the extracellular matrix of the cartilage was fully digested. Subsequently, the chondrocyte cells underwent filtration using a mesh screen and were subjected to centrifugation at a speed of 1200 revolutions per minute for a duration of 10 min at a temperature of 4 °C. Next, the supernatant liquid was carefully eliminated, and the cartilage in its pure form was subjected to washing using the phosphate-buffered saline (PBS) with the CAS number 10010-023. Subsequently, the cartilage was subjected to another round of centrifugation. The cells were transferred to a fresh dish and cultured in complete DMEM (Dulbecco’s modified Eagle’s media; CAS No.: 12100-046) at a temperature of 37 °C in a CO_2_ incubator with a 5% concentration of CO_2_.

#### 2.2.2. Cell Cultures

The SW1353 PRCs and RAW264.7 (American Type Culture Collection, Rockville, MD, USA) cells were cultured in Dulbecco’s Modified Eagle Medium (DMEM, 100 U/mL penicillin), streptomycin (100 μg/mL; CAS No.:15140-122), 10% fetal bovine serum, and 1% L-glutamine (CAS No.:25030-081) from Gibco BRL (Grand Island, NY, USA) and incubated at 37 °C in a humidified incubator containing 5% CO_2_ (DMEM, CAS No.: 12100-046; FBS, CAS No.: 26140-079; Penicillin-Streptomycin, CAS No.:15140-122; L-glutamine, 25030-081). The chosen PRC cell line third–fifth passage cells were used for whole experiments.

#### 2.2.3. 3-(4,5-Dimethylthiazol-2-yl)-2,5-Diphenyltetrazolium Bromide (MTT) Assay

The RAW264.7 cells were seeded in a 96-well plate (4 × 10^5^ cells/mL), while SW1353 PRC cell lines were seeded into a 96-well plate (1 × 10^5^ cells/mL) to culture overnight. Different concentrations of sesamin were added to each well, and the RAW264.7 cells were treated with 500 ng/mL LPS (EC number: 297-473-0). The SW1353 and PRC cell lines were treated with 10 ng/mL IL-1β (MDL number: MFCD01094098) for 24 h. Afterward, the cells were incubated for another 4 h after adding 5 mg/mL MTT (CAS number: 57360-69-7). Then, the medium was removed, and the undissolved formazan was dissolved in isopropanol overnight. The plate was measured at 570 nm.

### 2.3. Animals

Male SD rats (180~220 g) were housed in a controlled environment at 25 ± 1 °C with sufficient food and water and kept on an alternating 12 h dark and light cycle. The animal experiments were approved by Taipei Medical University’s Ethical Regulations on Animal Research (approval No.: LAC-100-0043). The animal studies were carried out in accordance with the guidelines outlined in the Guide for the Care and Use of Laboratory Animals, as specified by the National Institutes of Health (publication No. 85-23).

#### 2.3.1. MIA-Induced OA Model

**Figure 2:**
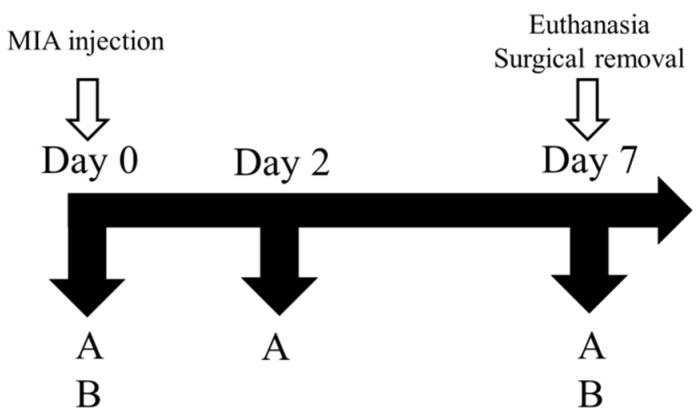
The timeline for monoiodoacetate (MIA)-induced osteoarthritis model. MIA: 50 μL (80 mg/mL). (A) Paw edema test. (B) Hind-limb weight-bearing test.

As depicted in Figure 2, the male Sprague-Dawley rats were categorized into four distinct groups, namely, control, positive control (PC), and sesamin groups. The induction of OA in male Sprague-Dawley rats was performed by administering an intra-articular injection of 80 μL of an MIA solution at a concentration of 80 mg/mL (Sigma) to the ankle joint. A 100 μL syringe was used for this purpose. The rats were randomly divided into six groups, with six rats assigned to each group. For a duration of 7 days, the sesamin groups were subjected to oral administration of sesamin at doses of 5, 10, and 20 mg/kg each day. The positive control group received a daily dosage of 20 mg/kg indomethacin for a duration of 7 days. The change in paw volume was measured using a plethysmometer (Ugo Basile, Comerio VA, Italy) on days 1 and 2 following the MIA injection. The hind limbs refer to the posterior appendages of an organism, often found in vertebrates, which are responsible for locomotion, and weight bearing was assessed by employing an in-capacitance tester equipped with a dual weight averager (Linton Instrumentation, Norfolk, UK). The measurements were conducted over a duration of 3 s and were performed in triplicate to ensure accuracy and reliability. In order to evaluate the ratio of weight distribution between the right hind paw (MIA injection side) and the left hind paw (control side), the following equation was employed:

$$\frac{\mathrm{mean}\;\mathrm{weight}\;\mathrm{of}\;\mathrm{the}\;\mathrm{right}\;\mathrm{hind}\;\mathrm{paw}}{\mathrm{mean}\;\mathrm{weight}\;\mathrm{of}\;\mathrm{the}\;\mathrm{left}\;\mathrm{hind}\;\mathrm{paw}}=\mathrm{Weight}-\mathrm{bearing}\;\mathrm{distribution}\;\mathrm{ratio}$$

#### 2.3.2. Histology of Cartilage and Immunohistochemistry

The rats’ right-side limbs were decalcified with a 10% Ethylene Diamine Tetraacetic Acid (EDTA) decalcifying solution for 7 days and maintained at 37 °C. The injured limbs were embedded in paraffin after decalcification. Serial sections of the samples were cut at a 5 μm thickness. These slides were stained with H&E stains. Under a microscope, pictures were taken for the OA analysis using a 10× eyepiece and a 20× objective.

### 2.4. In Silico Analysis (for Sesamin–RdRp and ACE2)

#### 2.4.1. Ligand Preparation

The three-dimensional structures (in the sdf file) of sesamin (*CID: 72307*) and sesamolin (*CID: 101746*) were from PubChem (chem.ncbi.nlm.nih.gov (accessed on 8 January 2022)). The sdf file of the compounds was converted to pdbqt using OpenBabel 2.4.1 (https://sourceforge.net/projects/openbabel/ (accessed on 8 January 2022)) [44].

#### 2.4.2. Protein Preparation and Validation

The binding site coordinates (listed in Table 1) of the proteins were from PDB (Protein Data Bank at https://www.rcsb.org/ (accessed on 8 January 2022)), and they were defined using Biovia Discovery Studio 2021 [45]. From Autodock Tools 4.2 [45], the polar hydrogens, Kollman charge, missing atom, and Autodock atom types were added for pre-processed protein. To validate the protein model, all prepared proteins were used in UCLA-DOE-LAB SAVES v6.0 (saves.mbi.ucla.edu (accessed on 8 January 2022)) and the Ramachandran plot server [46].

#### 2.4.3. Molecular Docking Analysis

To perform the docking analysis in AutoDock Vina, a computer system (Windows 11, AMD Ryzen 7 3700 U, 8.00 GB of RAM, and 2.30 GHz Radeon Vega Mobile Gfx) was used [49]. The grid parameters (binding site) used for the analyses are listed in Table 1.

#### 2.4.4. Molecular Dynamics Analysis

Promising compounds (with higher binding affinities) were subjected to a molecular dynamics simulation and compared with the positive control. The RMSF (root-mean-square fluctuation plot) and within 10 ns interaction model of the apoprotein and protein-ligand complexes were from CABS-Flex 2.0 (http://212.87.3.12/CABSflex2/submit (accessed on 8 January 2022)) [50].

#### 2.4.5. Pharmacophore, Drug-Likeness, and ADMET Screening

For drug-likeness and ADMET screening, the canonical SMILES of the compounds from PubChem were submitted to the ADMET 2.0 server (https://admetmesh.scbdd.com/ (accessed on 8 January 2022)). Mol2 files were converted from the sdf file in OpenBabel and were submitted to PharmaGist (http://bioinfo3d.cs.tau.ac.il/pharma/php.php (accessed on 8 January 2022)) [51] for pharmacophore modeling.

### 2.5. Statistical Analysis

Paw edema volume and MIA-induced paw edema data are expressed as means ± SDs. Variance among groups was evaluated using one- and two-factor ANOVAs (SPSS) and Duncan’s multiple range test. *p* < 0.05 was considered statistically significant (software: SPSS v.22 and Excel 2017).

## 3. Results and Discussion

### 3.1. Cell Toxicity

**Figure 3:**
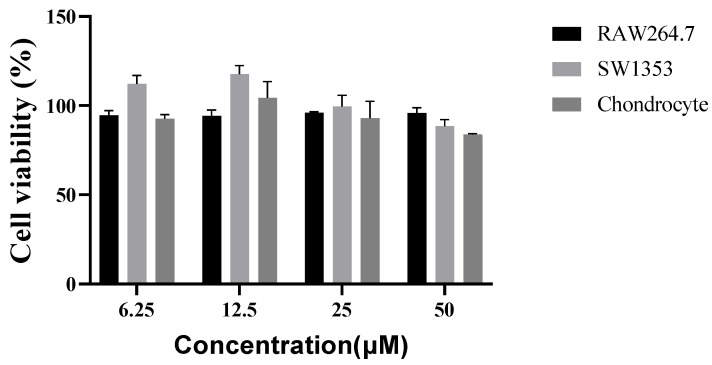
Cell viability, using the MTT assay, of three cell lines after 24 h of culture in the presence of different concentrations of sesamin.

After 24 h of treatment, the sesamin was not toxic to any cells at any dosage level (Figure 3).

### 3.2. Sesamin’s Anti-Inflammatory Effects

**Figure 4:**
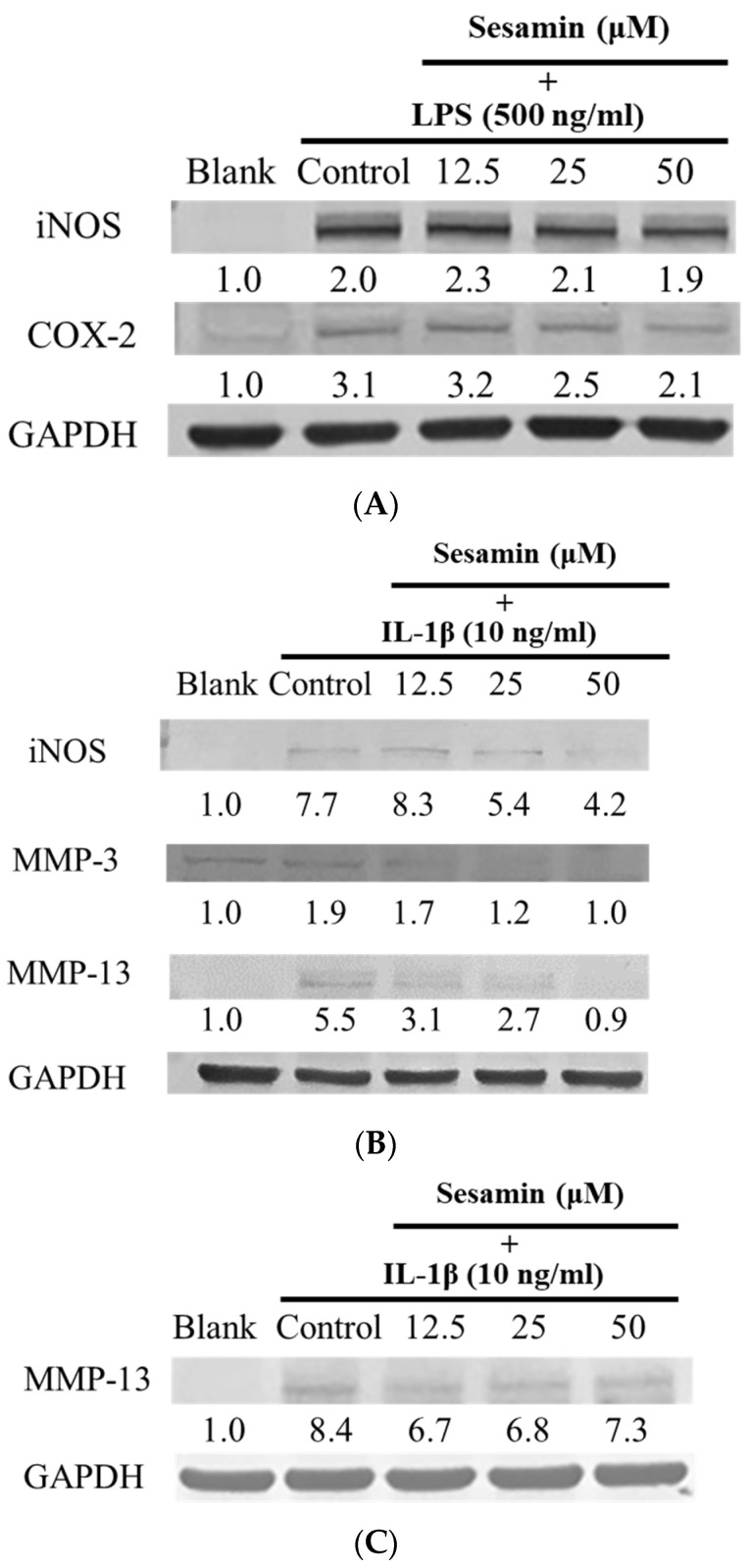
(**A**) Sesamin’s anti-inflammatory effect using LPS-induced RAW264.7 cells for 24 h. Quantitation of iNOS and COX-2 protein expression. Blank contained the medium only, control received LPS (500 ng/mL), and drug-treated groups received doses of 12.5, 25, and 50 μM sesamin + LPS (500 ng/mL). (**B**) Sesamin’s anti-inflammatory effect using IL-1β-stimulated chondrocyte cells for 24 h. Quantitation of iNOS, MMP-3, MMP-13 protein expression. Blank contained the medium only, control received IL-1β (10 ng/mL), and drug-treated groups received doses of 12.5, 25, and 50 μM sesamin + IL-1β (10 ng/mL). (**C**) Sesamin’s anti-inflammatory effect using IL-1β-induced SW1353 cells for 24 h. Quantitation of MMP-13 protein expression. Blank contained the medium only, control received IL-1β (10 ng/mL), and drug-treated groups received doses of 12.5, 25, and 50 μM sesamin + IL-1β (10 ng/mL).

iNOS and COX-2 are the most important mediators of inflammation-induced pain during the inflammatory process. RAW264.7 macrophage cells released NO when LPS was used to trigger the cells’ inflammatory response to measure the production of iNOS and COX-2. Sesamin treatment at doses between 12.5 and 50 μM demonstrated no cytotoxicity in any cell line, and the iNOS and COX-2 expressed in RAW264.7 cells are shown in Figure 4A.

Compared to the normal group, the arthritis patients had higher expression of MMP-3. The results showed that the treated group (sesamin doses of 12.5–50 μM) had no cytotoxicity, and the iNOS, MMP-3, and MMP-13 expressed in PRCs are shown in Figure 4B. Other dosages of 12.5–50 μM had no cytotoxicity, and the protein expression of MMP-13 in SW1353 cells is shown in Figure 4C.

### 3.3. Effects of Sesamin on Paw Edema Volume

**Figure 5:**
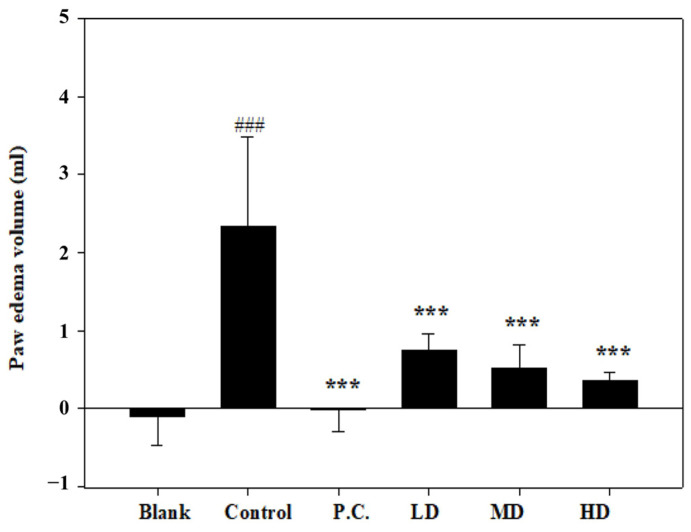
Paw edema volume was measured in MIA-injected rats on the second day. Positive control (P.C.) is indomethacin (2.5 mg/kg), LD: sesamin (5 mg/kg), MD: sesamin (10 mg/kg), HD: sesamin (20 mg/kg). Control group compared to a blank group: ### *p* < 0.0001. Sesamin and indomethacin groups compared to the control group: *** *p* < 0.001.

The paw edema volumes were determined and denoted as the baseline before the MIA injection. On the initial day of the experiment, the MIA injection caused edema in the right hind ankle of each rat. In comparison to the control group, the measured paw volumes increased significantly on the second day (Figure 5). In contrast, no swelling or volume change was observed in the positive control. Compared to the control group, sesamin reduced the paw edema volume in a dose-dependent manner. Thus, the 20 mg/kg sesamin treatment suppressed the swelling more than the lower dosages.

### 3.4. Effects of Sesamin on Hind-Limb Weight-Bearing Ratio

**Figure 6:**
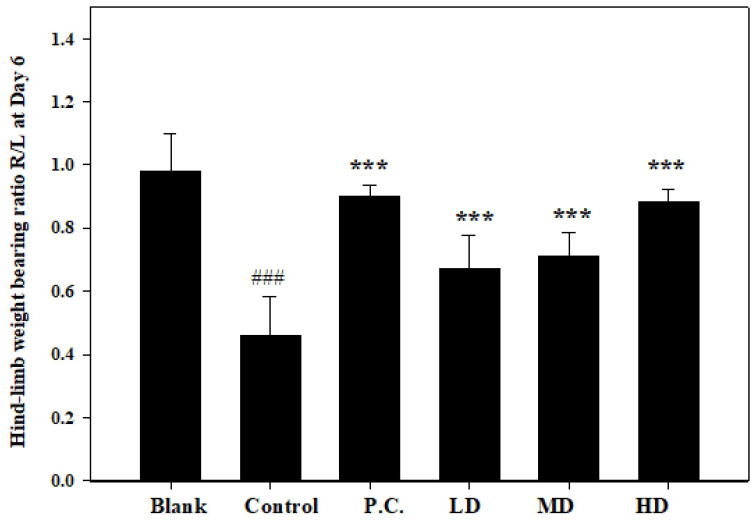
Paw edema volume was measured in MIA-injected rats on the second day. Positive control (P.C.) is indomethacin (2.5 mg/kg), LD: sesamin (5 mg/kg), MD: sesamin (10 mg/kg), HD: sesamin (20 mg/kg). Control group compared to blank group: ### *p* < 0.0001. Sesamin and indomethacin groups compared to control group: *** *p* < 0.001.

The evaluation of the weight-bearing ratio in rats with MIA-induced osteoarthritis demonstrated the anti-arthritic effect of sesamin as well as its effectiveness in suppressing inflammation. In this study, the right limb (injected with MIA) and the left limb were used as soreness indicators in the osteoarthritis of the ankle joint. The hind-limb weight-distribution ratios were determined on the seventh day using an in-capacitance tester. Daily oral administration of sesamin at doses of 5, 10, and 20 mg/kg (as shown in Figure 6) resulted in notable distinctions between the control group, positive control group, and the groups treated with sesamin. The groups administered sesamin exhibited a dose-dependent reduction in joint discomfort, with the treatment dosage of 20 mg/kg showing the most favorable outcomes. Additionally, there was no discernible disparity in the weight-bearing ratio between the treatment group receiving a dosage of 20 mg/kg and the positive control group. Therefore, the anti-arthritic efficacy of sesamin may potentially interact with the conventional treatment drug.

### 3.5. H&E Stain Assay

**Figure 7:**
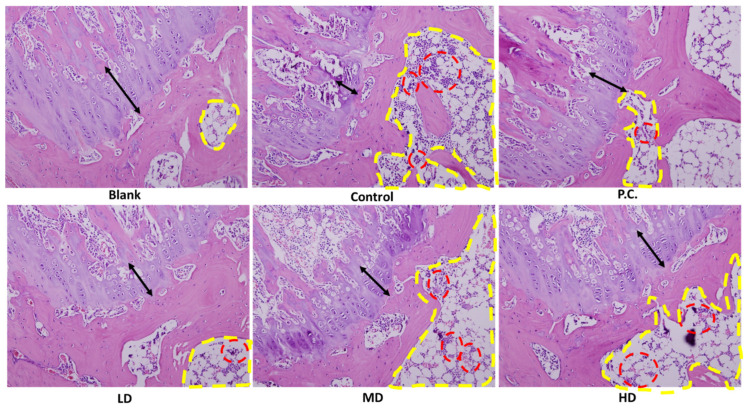
Cartilage histology and immunohistochemistry. H&E-stained knee joints. The positive control is indomethacin (2.5 mg/kg), LD: sesamin (5 mg/kg), MD: sesamin (10 mg/kg), HD: sesamin (20 mg/kg). The yellow circles represent the presence of cavities, while the red circles indicate the grouping of cells exhibiting intense staining. The black arrow indicates the existence of cartilage.

As shown in Figure 7, the cavity, delineated by the yellow dotted line, was initially observed, followed by the subsequent observation of the region enclosed by the red dotted line. Clusters of cells (lymphocytes) and deep stains were observable in the given context. In response to an inflammatory response, lymphocytes proliferate and aggregate. Furthermore, the black double arrow serves as an indication of the presence of cartilage. The cartilage area in the blank group exhibited a tight and orderly arrangement with a somewhat wide distribution. The chondrocytes in the inner part were well organized with an intact arrangement. In contrast, the blank group’s cartilage area did not have any blood vessels, which meant it was very healthy, and the chondrocytes were also broken. The results indicate that the low-dose group exhibited a slow recovery in the cartilage region upon exposure to sesamin. Furthermore, the chondrocytes in this group displayed a generally complete and orderly arrangement. An observation could be made that with the escalation in dosage, the cartilage area exhibited a restoration characterized by a taut and orderly arrangement. The chondrocytes exhibited a high degree of organization and uniformity, as depicted in Figure 7.

### 3.6. Anti-Viral Activity Screening

For this study, the RdRp of SARS-CoV-2 (*PDB: 7BTF*) and human ACE2 (*PDB: 1R42*) were selected to screen the potential anti-viral activity of sesamin. Table 2 shows processed proteins that were validated with different algorithms (Verify3D, ERRAT, and Ramachandran plot) to ensure the model’s validity before the docking analysis.

### 3.7. Molecular Docking Analysis

**Figure 8:**
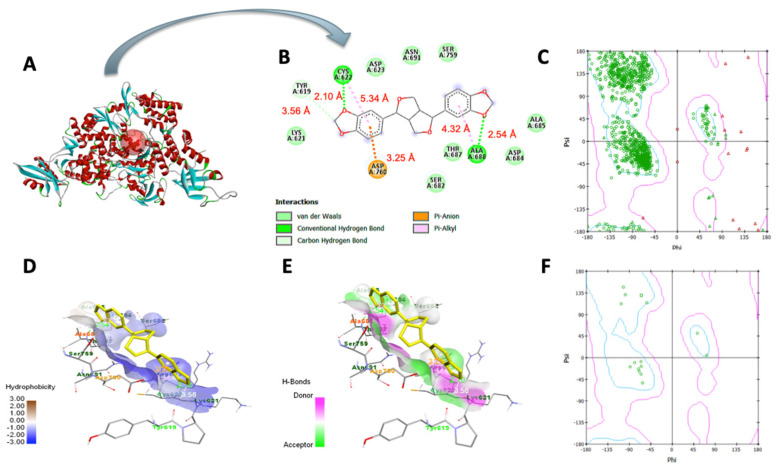
Molecular docking of sesamin with COVID−19 RdRp protein: (**A**) viral RdRp−sesamin complex, (**B**) two–dimensional interaction display, (**C**) Ramachandran plot of unbound protein, (**D**) hydrophobic and (**E**) hydrophilic properties of binding pocket, (**F**) Ramachandran plot of ligand−interacting amino acid residues.

**Figure 9:**
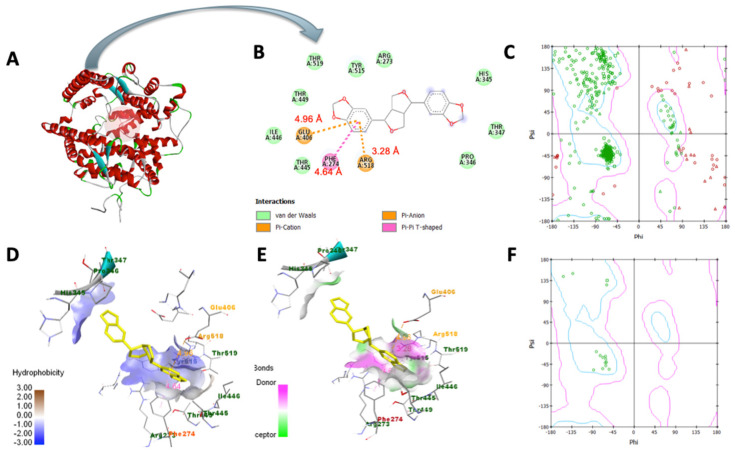
Molecular docking of sesamin with ACE2: (**A**) ACE2−sesamin complex, (**B**) two−dimensional interaction display, (**C**) Ramachandran plot of unbound protein, (**D**) hydrophobic and (**E**) hydrophilic properties of binding pocket, (**F**) Ramachandran plot of ligand−interacting amino acid residues.

**Figure 10:**
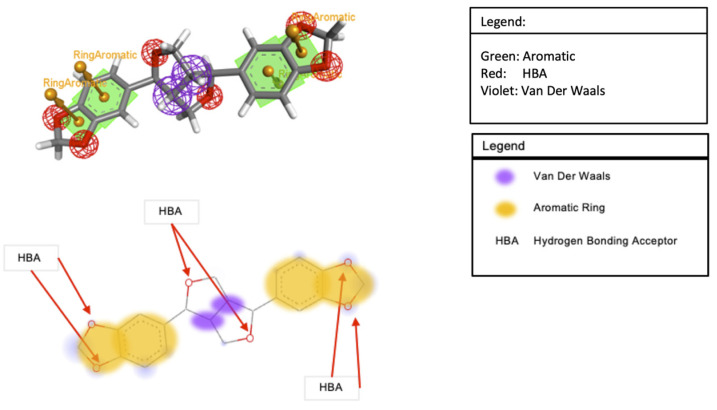
Pharmacophore descriptor of sesamin.

In Table 3, sesamin exhibited a better binding affinity with the target protein than the control. Hydrogen bonding and van der Waals, pi–anion, pi–cation, pi–Alkyl, and pi–pi T-shaped interactions were the non-bonding interaction forces observed between the interacting amino acids and sesamin. As observed in Figure 8B and Figure 9B, van der Waals forces dominated the protein–ligand association, which was expected given that both proteins’ binding pockets (Figure 8D and Figure 9D) are very hydrophobic [52]. Moreover, the pharmacophore descriptors (Figure 10) of sesamin also impact the interacting forces of the protein–ligand association. For example, the aromatic rings of sesamin were involved in pi–alkyl (with Cys 622 and Ala 687 of viral RdRp), pi–anion (with Asp 760 of viral RdRp and Glu 406 of ACE2), pi–cation (with Arg 518 of the ACE2 protein), and pi–pi T-shaped (with Phe 214 of the ACE2 protein) interactions [53,54]. The hydrogen-bonding acceptors of sesamin interacted with Cys 622 and Ala 688 in the binding pocket (Figure 8E) of viral RdRp via hydrogen bonding interactions [55,56]. Together, 13 amino acids (5 helices, 5 turns, and 3 coils) of viral RdRp and 12 amino acids (7 helices, 3 coils, and 1 sheet) of the ACE2 protein interact with sesamin during ligand binding.

### 3.8. Molecular Dynamics Simulation

**Figure 11:**
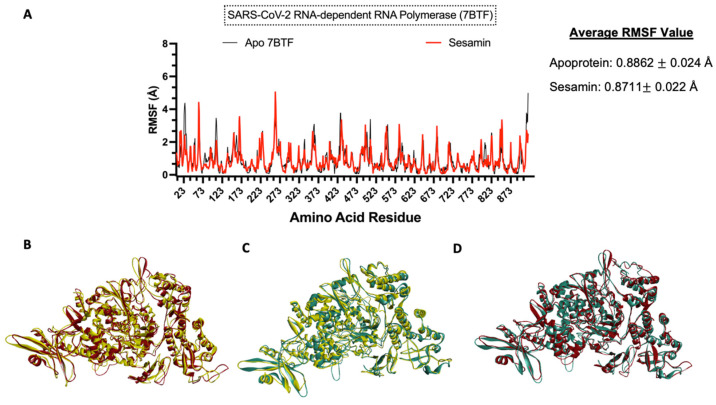
Molecular dynamics analysis of sesamin−SARS-CoV-2 RdRp complex. (**A**) Amino acid residues of apoprotein RMSF values and ligand-bound protein. Three-dimensional models of (**B**) changes in apoprotein in 0 ns (yellow) and 10 ns (red) and (**C**) changes in ligand-bound protein in 0 ns (yellow) and 10 ns (teal) and (**D**) overlap model of apoprotein (red) and ligand-bound protein (teal).

**Figure 12:**
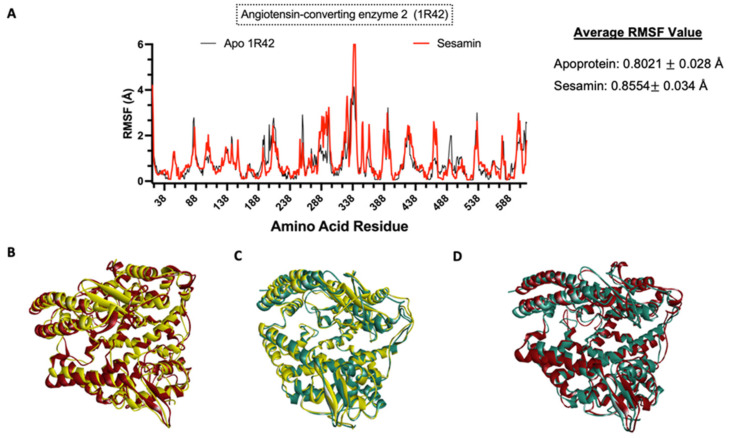
Molecular dynamics analysis of sesamin–human ACE2 complex. (**A**) Amino acid residues of apoprotein RMSF values and ligand-bound protein. Three-dimensional models of (**B**) changes in apoprotein in 0 ns (yellow) and 10 ns (red) and (**C**) changes in ligand-bound protein in 0 ns (yellow) and 10 ns (teal) and (**D**) overlap model of apoprotein (red) vs. ligand-bound protein (teal) at 10 ns.

To understand the impact of ligand binding on the protein structure, molecular dynamics simulation was performed on the apoprotein and the protein–sesamin complexes (Figure 11B and Figure 12B) and the protein–ligand complexes (Figure 11C and Figure 12C). Based on the average RMSF value, the COVID-19 RdRp protein was stiffer after ligand binding. This behavior was also observed in remdesivir, which inhibits the viral replication process. Therefore, the spread of the virus and the viral load would be decreased [57]. At the same time, the ACE2 protein became more flexible after ligand binding.

### 3.9. Drug-Likeness and ADMET Screening

**Figure 13:**
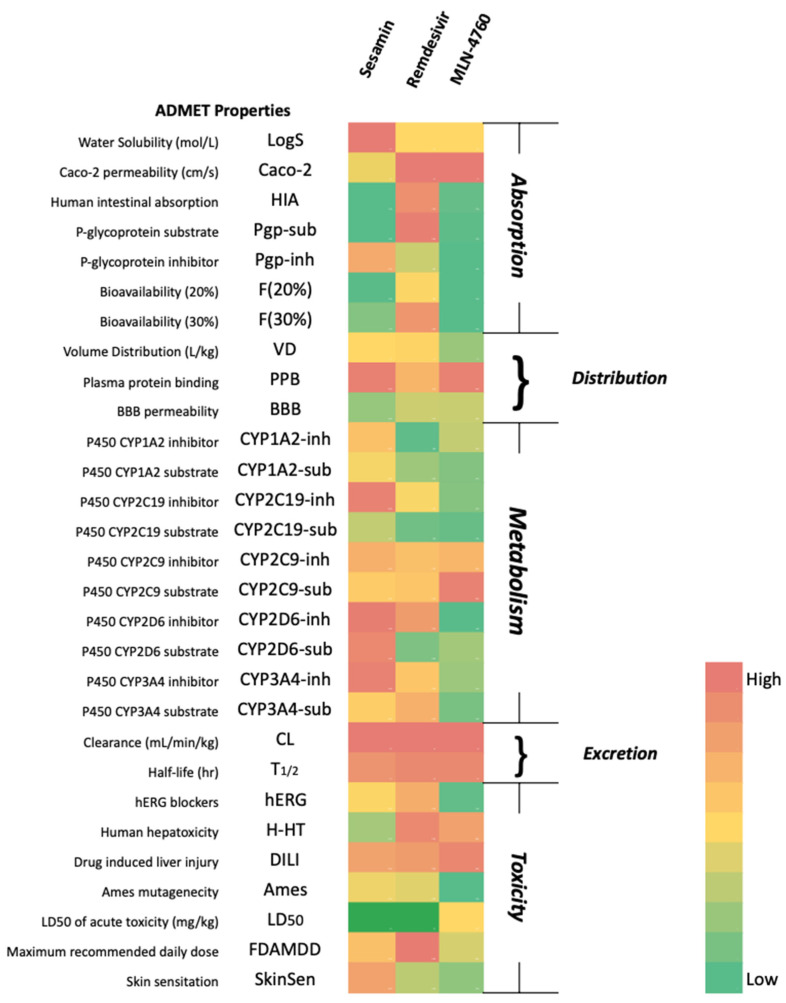
Pharmacokinetic properties of the compounds.

Earlier analyses suggested that sesamin has great potential to be developed as an anti-SARS-CoV-2 drug by acting as an RdRp and ACE2 inhibitor. Therefore, the drug-likeness and ADMET properties of sesamin were studied and compared with the control. As shown in Table 4 and Figure 13, sesamin passed the criteria for Lipinski’s rule of five and had less toxicity than the control [58]. Based on the profile, sesamin may be metabolized by the P450 enzymes present in the liver (first-pass metabolism) before reaching the target site [59]. Just like remdesivir, a prodrug may be considered to overcome the first-pass effect and further enhance the bioavailability of the drug [60,61].

## 4. Discussion

According to research findings, inflammation has been identified as the primary factor contributing to mortality in COVID-19 patients. The cytokines include TNF, NF-κB, IL-1β, IL-6, IL-8, etc. The inflammatory macrophages express kinds of cytokines that trigger downstream inflammation pathways, resulting in inflammation and damage to organs [62,63]. As a result of tissue variation, matrix metalloproteinases (MMPs) are classified as the mediators of organ damage and inflammation. Several clinical studies have observed a correlation between the blood MMP level in COVID-19 patients and the disease severity, which MMP expression increases as the disease progresses [64,65]. Therefore, we designed the in vivo and in vitro studies to examine the enduring impact of COVID-19 on the progression of osteoarthritis and inflammation, specifically focusing on the expression of MMPs.

Based on the in vitro investigation, sesamin induces iNOS and COX-2 as well as MMP-3 and MMP-13. Previous studies have demonstrated that matrix metalloproteinases (MMPs) play a crucial role in the degradation of the extracellular matrix of cartilage [24,26]. Thus, inhibiting MMP-13 plays an important role in osteoarthritis treatment. In human articular chondrocytes (HACs) cultured from papain-induced OA rats, sesamin reversibly affected the expression of MMP-1, MMP-3, and MMP-13 without affecting aggrecanase activity. [66] Interestingly, sesamin reduces IL-1β-induced MMP expression and protein translation, inhibits inflammatory gene expression (e.g., NF-kB pathway suppression), reverses the synergistic effect caused by OSM (oncostatin M) and IL-1β, and stops type II collagen and proteoglycan degradation. In addition, a clinical investigation using samples collected from 12 patients undergoing knee replacement surgery revealed that sesamin activated the Nrf2 signaling pathway in chondrocytes affected by osteoarthritis (OA), resulting in an upregulation of HO-1 protein expression [67].

After validating the processed protein, a docking analysis was performed to examine sesamin’s binding affinity with the target proteins. Also, remdesivir [58] and MLN-4760 [68] were selected as positive controls for the analysis. Remdesivir is a nucleotide prodrug of an adenosine analog that attaches to viral RNA polymerase and inhibits viral replication [69,70]. Angiotensin-converting enzyme 2 (ACE2) is an enzyme in the body. The COVID-19 (SARS-CoV-2) spike S1 protein can bind to the ACE-2 receptor, thus allowing viral admittance into the target cell, leading to a risk of damage to the respiratory system, liver, heart, intestines, and kidneys and thrombus formation [71].

We found that the ACE2 protein interacts with sesamin during ligand binding and becomes more flexible after ligand binding. In a study conducted by Hubbard et al. [56], it was stated that hydrogen bonding forces have a significant impact on the secondary structure of a protein. Unlike the sesamin–RdRp complex, a hydrogen bonding interaction was not detected in the sesamin–ACE2 complex. In addition, the RMSF plot (Figure 12A) of the complex shows that the interacting amino acids and amino acids around the binding pocket had a larger fluctuation than the apoprotein. Such conditions were also observed in some compounds that were observed to have high binding affinities and longer residence times, as reported by Amaral et al. [72].

## 5. Conclusions

COVID-19 (SARS-CoV-2) may trigger the overproduction of pro-inflammatory cytokines and induce a hyper-inflammatory response, leading to organ damage and other disease symptoms known as “long-term COVID”. In this study, we used macrophage cell lines to simulate the inflammatory response and used PRCs and SW1353 chondrocyte cell lines to mimic the inflammatory response of joint tissue. Using RAW264.7 macrophages stimulated with LPS, sesamin inhibited COX-2 and iNOS expression in in vitro studies. For the chondrocyte mediators of SW1353, chondrosarcoma, and PRCs, as observed, sesamin could inhibit the iNOS, MMP-3, MMP-13 expression of PRCs and the MMP-13 expression of SW1353 cells.

Furthermore, the anti-arthritic property of sesamin was assessed through the MIA-induced OA rat model, revealing that sesamin could reduce paw swelling and alleviate joint discomfort in a dose-dependent manner. The results indicated that a high dose of sesamin would hinder the progression of OA. Therefore, *Sesamum Indicum* L. possesses a potential nutritional supplement for inflammatory diseases related to arthritis. Based on molecular simulation and ADMET screening, sesamin seems to be a promising anti-viral lead compound with a higher binding affinity against ACE2 (than COVID-19/SARS-CoV-2 RdRp). Hence, sesamin could limit virion entry and the whole replication process. Therefore, we suggest that sesamin is a promising lead for anti-viral drugs with immunomodulatory capabilities.

## Figures and Tables

**Table 1:** Protein target for docking analysis.

Target	PDB Code	Grid Parameters of the Binding Site		Reference
		Coordinates	Size	
Viral RdRp	7BTF	x = 127.0598	x = 14	[47]
		y = 124.6167	y = 14	
		z = 128.7631	z =14	
ACE2	1R42	x = 52.292033	x = 10	[48]
		y = 62.932978	y = 10	
		z = 29.089189	z = 10	

**Table 2:** Protein validation.

Target Protein	Verify3D ≥ 80%	ERRAT ≥ 50%	Ramachandran Plot (Amino Acid in the Preferred Region) ≥ 95%
SARS-CoV-2 Viral RdRp	82.44	89.264	99.551
Human ACE2	92.13	95.068	98.881

**Table 3:** Binding energies of the ligands.

	Ligand	Sesamin	Remdesivir	MLN-4760
**Binding Energy (kcal/mol)**	SARS-CoV-2 RdRp	−6.6	−6.1	n.a.
	Human ACE2	−5.1	n.a.	−4.8

n.a.: not applicable.

**Table 4:** Drug-likeness analysis of the compounds.

Compound	Sesamin	Remdesivir	MLN-4760
Molecular Formula	C_20_H_18_O_6_	C_27_H_35_N_6_O_8_P	C_19_H_23_Cl_2_N_3_O_4_
Molecular Weight (Da) ≤ 500	354.11	602.60	428.30
Log *p* ≤ 5	4.051	1.761	0.836
HBA *p* ≤ 5	0	5	3
HBD *p* ≤ 10	6	14	7
TPSA (Å^2^) ≤ 140 Å^2^	55.38	204.28	104.45

HBA: hydrogen-bond acceptor; HBD: hydrogen-bond donor; Log **p**: lipophilicity; TPSA: total polar surface area.

## Data Availability

The data are available from the authors (see given emails).
